# Baicalein and Neohesperidin Dihydrochalcone Improve Post-Thaw Sperm Quality and Modulate the Semen Supernatant Metabolome in Qinchuan Bulls

**DOI:** 10.3390/ani16203210

**Published:** 2026-10-12

**Authors:** Benshun Yang, Zhipeng Zhang, Song Gao, Ruiyang Zheng, Linsen Zan, Wucai Yang

**Affiliations:** College of Animal Science and Technology, Northwest A&F University, Yangling 712100, China

**Keywords:** baicalein, neohesperidin dihydrochalcone, semen cryopreservation, metabolomics, oxidative stress

## Abstract

Oxidative stress during cryopreservation compromises post-thaw sperm quality in bulls. This study evaluated the effects of baicalein (BAI), neohesperidin dihydrochalcone (NHDC) and their combined supplementation on Qinchuan bull sperm cryopreservation and the associated metabolic responses. Concentration screening identified 40 μg/mL BAI and 30 μg/mL NHDC for subsequent experiments. Compared with the control, BAI, NHDC and BAI + NHDC increased post-thaw sperm motility, plasma membrane integrity, acrosome integrity, adenosine triphosphate (ATP) content and mitochondrial membrane potential (MMP). The supplemented groups also showed lower reactive oxygen species (ROS) and malondialdehyde (MDA) levels; higher activities of superoxide dismutase (SOD), catalase (CAT), and glutathione peroxidase (GSH-Px); and higher total antioxidant capacity (T-AOC). The combined treatment showed higher total motility, plasma membrane integrity, and acrosome integrity than either single treatment. Untargeted metabolomics identified 96, 123 and 197 differential metabolites in the BAI, NHDC and BAI + NHDC groups, respectively. Differential metabolites from all three treatments were enriched in lipid-related pathways, including glycerophospholipid metabolism and linoleic acid metabolism. Metabolites identified in the BAI + NHDC group were additionally enriched in nucleotide metabolism, purine metabolism, and ubiquinone-related pathways. BAI × NHDC interaction analysis identified 200 metabolites showing non-additive responses, with enrichment in lipid-related pathways, including glycerophospholipid metabolism and linoleic acid metabolism. Together, BAI and NHDC improved post-thaw sperm quality in Qinchuan bulls, accompanied by changes in oxidative status, mitochondrial function, and lipid- and energy-related metabolites.

## Simple Summary

Cryopreservation of bull semen enables the long-term preservation and distribution of valuable genetic resources, but the freezing and thawing process can damage sperm cells. We evaluated two plant-derived antioxidant compounds, baicalein and neohesperidin dihydrochalcone, alone and in combination, to improve the quality of frozen–thawed semen from Qinchuan bulls. Both compounds improved sperm motility, membrane integrity, energy metabolism and antioxidant capacity after thawing. The combined treatment showed higher total motility, plasma membrane integrity, and acrosome integrity than either single treatment. Untargeted metabolomics of semen supernatant revealed treatment-associated alterations in metabolic pathways. These findings suggest that baicalein and neohesperidin dihydrochalcone may be useful additives for improving bull semen preservation and supporting the use of valuable Qinchuan cattle genetic resources.

## 1. Introduction

Artificial insemination is widely used in cattle breeding, and semen cryopreservation is essential for the long-term storage and distribution of superior genetic resources. However, the freeze–thaw process can reduce sperm motility, plasma membrane integrity, acrosome integrity and mitochondrial function, thereby impairing post-thaw sperm quality [1,2]. These impairments are mainly associated with cold shock, osmotic stress, ice crystal formation and oxidative stress during cryopreservation [3]. Therefore, improving antioxidant protection in semen extenders remains an important strategy to protect post-thaw sperm quality.

Oxidative stress is a major cause of sperm cryodamage. Bull sperm membranes are rich in polyunsaturated fatty acids and highly susceptible to lipid peroxidation, while sperm possess limited endogenous antioxidant capacity [4,5]. Excessive reactive oxygen species (ROS) can damage membrane lipids, proteins and mitochondrial function, leading to reduced motility and structural integrity after thawing [6,7]. Accordingly, various antioxidant additives have been evaluated in semen extenders to reduce oxidative injury and maintain sperm function during cryopreservation. In recent years, plant-derived antioxidants have also been tested in semen freezing extenders, including resveratrol [8], lycopene [9], quercetin [10], curcumin [11] and rosemary extract [12].

Baicalein (BAI) is a flavone compound with antioxidant, anti-inflammatory and mitochondrial protective properties. Previous studies have shown that BAI can reduce oxidative injury and improve cell survival under stress conditions [13]. Neohesperidin dihydrochalcone (NHDC) is a citrus-derived dihydrochalcone compound with free-radical-scavenging and antioxidant activities [14,15]. Although BAI and NHDC differ in chemical structure and source, both have antioxidant potential and may protect sperm against freeze–thaw-induced oxidative damage. Combined antioxidant supplementation can provide complementary protection by targeting different oxidative pathways, and has been shown to improve post-thaw semen quality [16,17]. However, the effects of BAI and NHDC, alone or in combination, have not been fully evaluated in bull semen cryopreservation.

In addition to conventional sperm quality indicators, metabolomic analysis provides a useful approach to characterize metabolic changes associated with sperm cryodamage and extender supplementation [18,19]. Changes in lipid metabolism, amino acid metabolism, nucleotide metabolism and energy-related pathways may reflect alterations in membrane stability, oxidative status and mitochondrial function after thawing [20,21]. Therefore, metabolomics can help determine whether antioxidant supplementation alters the metabolic environment of post-thaw semen and whether combined additives induce responses different from those caused by single additives.

The objective of this study was to evaluate the effects of BAI, NHDC and BAI + NHDC on post-thaw sperm quality in Qinchuan bulls. Sperm motility, plasma membrane integrity, acrosome integrity, adenosine triphosphate (ATP) content, mitochondrial membrane potential (MMP), oxidative status and antioxidant capacity were assessed. Untargeted metabolomics was further used to characterize metabolic changes in post-thaw semen supernatant and to explore the non-additive metabolic response associated with combined BAI and NHDC supplementation.

## 2. Materials and Methods

### 2.1. Ethics Statement

All experimental procedures were approved by the Experimental Animal Center of Northwest A&F University (NWAFUCAST2018-168).

### 2.2. Semen Collection and Quality Assessment

Semen was collected from six healthy Qinchuan bulls aged 3–5 years at the National Beef Cattle Improvement Center of Northwest A&F University, Yangling, Shaanxi, China. Bulls were maintained under the same feeding and management conditions and had normal reproductive performance. Semen was collected twice weekly using an artificial vagina.

Total motility, sperm concentration, and morphology were assessed immediately after collection using the SQA-Vb analyzer (MES, Encino, CA, USA). Only ejaculates with total motility ≥ 70%, sperm concentration ≥ 6 × 10^8^ sperm/mL and abnormal sperm morphology ≤ 15% were used [22].

### 2.3. Additive Treatments

Three independent experiments were conducted using separate semen collection and pooling batches to determine the effective concentrations of BAI (Jskchem, Shanghai, China) and NHDC (Jskchem, Shanghai, China) and to evaluate their combined effects on post-thaw sperm quality. In Experiment 1, BAI was added to the semen diluent at final concentrations of 0, 20, 40, 60, and 80 μg/mL. In Experiment 2, NHDC was added at 0, 10, 20, 30, and 40 μg/mL. Based on the screening results, 40 μg/mL BAI and 30 μg/mL NHDC were selected for subsequent experiments. In Experiment 3, four treatments were established: control (CON), BAI (40 μg/mL), NHDC (30 μg/mL), and BAI + NHDC (40 μg/mL BAI and 30 μg/mL NHDC). BAI was dissolved in dimethyl sulfoxide (DMSO), whereas NHDC was dissolved in water. DMSO concentrations were matched across groups within Experiments 1 and 3 (≤ 0.5%, *v*/*v*); no DMSO was added in Experiment 2.

For each experiment, semen collected from the six bulls was pooled and divided among the corresponding treatment groups, which were supplemented and cryopreserved separately. This entire collection, pooling, treatment, and cryopreservation procedure was performed on six separate occasions, providing six pooled-semen batch replicates per treatment (n = 6).

### 2.4. Semen Freezing and Thawing

Fresh semen was diluted using a one-step dilution method by mixing with preheated (37 °C) semen diluent (IMV, Optidyl, 020996, L’Aigle, France) to achieve a final sperm concentration of 5 × 10^7^/mL. The diluted semen was equilibrated at 4 °C for 4 h, loaded into 0.25 mL straws, sealed, and frozen in liquid nitrogen vapor. The straws were placed 5 cm above the surface of liquid nitrogen for 15 min and then plunged into liquid nitrogen for storage. After at least 7 d of storage, the straws were thawed in a 37 °C water bath for 30 s and immediately subjected to post-thaw evaluation. Post-thaw total motility, progressive motility, and instrument-reported velocity (μm/s) were assessed using the SQA-Vb analyzer.

### 2.5. Assessment of Plasma Membrane and Acrosome Integrity

Plasma membrane integrity was assessed using the hypo-osmotic swelling test [23]. The hypo-osmotic solution contained 0.6756 g fructose and 0.3676 g glucose in 50 mL double-distilled water, and its osmolarity was experimentally measured as 150 mOsm/L. Thawed semen (100 μL) was mixed with 1 mL hypo-osmotic solution and incubated at 25 °C for 30 min. Sperm with curled or swollen tails were considered membrane-intact. At least 100 sperm cells were counted per sample, without correction for baseline tail coiling in iso-osmotic medium.

Acrosome integrity was assessed using fluorescein isothiocyanate-conjugated peanut agglutinin (FITC-PNA) staining. Semen smears were air-dried, fixed in anhydrous methanol for 10 min, stained with FITC-PNA at 37 °C for 30 min in the dark, washed with phosphate-buffered saline and observed under a fluorescence microscope. Sperm with uniform green fluorescence in the acrosomal region were classified as acrosome-intact. At least 100 sperm cells were counted per sample. Illustrations of acrosome and plasma membrane integrity are provided in Figure S1.

### 2.6. Determination of ATP Content and MMP

ATP content was measured spectrophotometrically using a commercial ATP assay kit (Solarbio, Beijing, China). MMP was assessed using a JC-1 assay kit (Beyotime, Shanghai, China). Red and green fluorescence intensities were measured using a multimode microplate reader (Infinite M200 PRO; Tecan, Männedorf, Switzerland), and MMP was expressed as the red/green fluorescence ratio. Equal volumes of thawed semen were washed, and the resulting sperm pellets were processed using the same volume of extraction buffer. Assays were performed according to the manufacturers’ instructions.

### 2.7. Assessment of Oxidative Status and Antioxidant Capacity

Post-thaw oxidative status and antioxidant capacity were evaluated using commercial assay kits (Solarbio, Beijing, China). ROS was assessed using the 2′,7′-dichlorodihydrofluorescein diacetate (DCFH-DA) fluorescent probe, with fluorescence measured using a multimode microplate reader (Infinite M200 PRO; Tecan, Männedorf, Switzerland). malondialdehyde (MDA), superoxide dismutase (SOD), catalase (CAT), glutathione peroxidase (GSH-Px), and total antioxidant capacity (T-AOC) were measured spectrophotometrically in washed sperm lysates. All assays were performed and results calculated according to the manufacturers’ instructions.

### 2.8. Untargeted Metabolomic Analysis

For metabolomic analysis, thawed semen samples from the CON, BAI, NHDC, and BAI + NHDC groups were centrifuged at 3000× *g* for 15 min at 4 °C to remove spermatozoa, and the resulting semen supernatant was collected and stored at −80 °C. Six semen supernatant samples per group, each obtained from a separate collection and cryopreservation batch, were analyzed (n = 6).

After thawing on ice and vortexing for 10 s, 100 μL semen supernatant was mixed with 100 μL internal-standard extraction solution (acetonitrile:methanol = 1:4, *v*/*v*), vortexed for 3 min, and centrifuged at 12,000× *g* for 10 min at 4 °C. A 150-μL aliquot of the supernatant was held at −20 °C for 30 min and centrifuged again at 12,000× *g* for 3 min at 4 °C; 120 μL was transferred for liquid chromatography–mass spectrometry (LC–MS) analysis. Equal aliquots of all samples were pooled to prepare quality-control samples. Chromatographic separation was performed using a Vanquish ultrahigh-performance liquid chromatography system coupled to a Q Exactive HF-X mass spectrometer (Thermo Scientific, Waltham, MA, USA) and an ACQUITY Premier HSS T3 column (1.8 μm, 2.1 × 100 mm; Waters Corporation, Milford, MA, USA). The mobile phases were 0.1% formic acid in water (A) and 0.1% formic acid in acetonitrile (B); the column temperature was 40 °C, the flow rate was 0.4 mL/min, and the injection volume was 3 μL. Data were acquired in positive and negative electrospray ionization modes over *m*/*z* 70–1000 at an MS1 resolution of 60,000, with spray voltages of 3.8 and 3.4 kV, respectively, and stepped collision energies of 30, 40, and 50 V. Raw files were converted to mzML format using ProteoWizard v3.0.7414 and processed in XCMS for peak extraction, alignment, and retention-time correction. Features missing from more than 50% of samples within a group were removed; remaining missing values were imputed using K-nearest neighbors or one-fifth of the minimum value according to the proportion of missingness, and peak areas were corrected by support vector regression. Metabolites were annotated by accurate-mass, retention-time, and MS/MS spectral matching, using mass tolerances of 25 ppm (MS1) and 50 ppm (MS2), a retention-time matching tolerance of 6 s, a minimum MS/MS match score of 0.3, and a minimum composite score of 0.5. According to the Metabolomics Standards Initiative (MSI), annotations supported by MS/MS library matching, without confirmation using authentic standards under the same analytical conditions, were classified as level 2 (putatively annotated compounds).

No targeted exclusion of BAI, NHDC, or features potentially corresponding to their isomers or adducts was performed during metabolomic data processing. Principal component analysis (PCA) was performed after log_2_ transformation and zero-centering. Partial least-squares discriminant analysis (PLS-DA) was conducted after log_2_ transformation and unit-variance scaling using MetaboAnalystR v1.0.1, and model robustness was evaluated by 200 permutation tests. Differential metabolites were selected using variable importance in projection (VIP) > 1, false discovery rate (FDR) < 0.05, and |log_2_FC| ≥ 1, followed by annotation using the Kyoto Encyclopedia of Genes and Genomes (KEGG) Compound database. KEGG pathway enrichment was performed using a one-sided hypergeometric test, with all detected metabolites successfully mapped to KEGG used as the background set. Pathways were ranked according to unadjusted *p* values, with *p* < 0.05 used as an exploratory enrichment threshold. No multiple-testing correction was applied to the pathway enrichment *p* values.

### 2.9. BAI × NHDC Interaction Analysis

A two-way analysis of variance (ANOVA) was performed for each metabolite, with BAI, NHDC, and BAI × NHDC as fixed factors. The interaction effect was calculated as follows:

Interaction effect = mean (BAI + NHDC) − mean (BAI) − mean (NHDC) + mean (CON).

*p* values for the interaction term were adjusted using the Benjamini–Hochberg FDR method. Metabolites with an FDR-adjusted interaction *p* < 0.05 were retained as interaction-associated metabolites. Those with positive interaction effects were classified as positive non-additive metabolites, whereas those with negative interaction effects were classified as negative non-additive metabolites. The interaction analysis was performed using log_2_-transformed metabolite intensities.

### 2.10. Statistical Analysis

Sperm quality and biochemical data from each experiment were analyzed separately using one-way ANOVA followed by Duncan’s multiple range test in SPSS v27.0. Homogeneity of variance was assessed using Levene’s test. Results are presented as mean ± standard deviation (SD). *p* < 0.05 was considered significant, and different lowercase letters within the same column indicate significant differences among treatments.

## 3. Results

### 3.1. Effects of BAI, NHDC, and BAI + NHDC on Post-Thaw Sperm Motility

The effects of BAI and NHDC treatments on the motion characteristics of frozen–thawed bull sperm are presented in Table 1. Compared with the control group, BAI treatment significantly increased total motility, progressive motility, and sperm velocity (*p* < 0.05), with the highest values observed at 40 μg/mL BAI. Similarly, NHDC treatment significantly enhanced sperm motion characteristics compared with the control group (*p* < 0.05). Among the tested concentrations, 30 μg/mL NHDC showed the most pronounced effect.

Based on the most effective concentrations identified above, the combined effects of BAI and NHDC were further evaluated. Compared with the control group, BAI, NHDC, and BAI + NHDC treatments significantly improved total motility and progressive motility (*p* < 0.05). Progressive motility did not differ significantly among the three supplemented groups. The combined group showed significantly higher total motility than either single treatment, whereas sperm velocity did not differ significantly from NHDC alone.

### 3.2. Post-Thaw Structural Integrity and Mitochondrial Function

The effects of BAI, NHDC, and their combination on post-thaw sperm structural integrity and mitochondrial function are shown in Table 2. Compared with the control group, BAI treatment significantly improved plasma membrane integrity, acrosome integrity, ATP content, and MMP of frozen–thawed spermatozoa (*p* < 0.05). Among the tested concentrations, 40 μg/mL BAI exhibited the most pronounced protective effect. Similarly, NHDC treatment significantly enhanced sperm structural integrity and mitochondrial function compared with the control group (*p* < 0.05). ATP content did not differ significantly among the NHDC-supplemented groups.

Based on the most effective concentrations identified above, the effects of combined BAI and NHDC treatment were further evaluated. Compared with the control group, BAI, NHDC, and BAI + NHDC treatments significantly increased membrane integrity, acrosome integrity, ATP content, and MMP (*p* < 0.05). The BAI + NHDC group showed significantly higher plasma membrane integrity and acrosome integrity than the NHDC group, whereas ATP content and MMP did not differ significantly from NHDC alone.

### 3.3. Post-Thaw Oxidative Status and Antioxidant Capacity

The effects of BAI, NHDC, and their combination on oxidative status and antioxidant capacity of post-thaw spermatozoa are presented in Table 3. Compared with the control group, BAI and NHDC treatments significantly reduced relative ROS and MDA levels while enhancing the activities of antioxidant enzymes, including SOD, CAT, and GSH-Px, as well as T-AOC (*p* < 0.05). Among the tested concentrations, 40 μg/mL BAI and 30 μg/mL NHDC exhibited the strongest antioxidant effects, which were consistent with the most effective concentrations identified in previous experiments.

Furthermore, the combined treatment of BAI and NHDC produced a greater improvement in antioxidant capacity compared with individual treatments. The BAI + NHDC group showed the lowest ROS and MDA levels and the highest antioxidant enzyme activities and T-AOC among all groups (*p* < 0.05), indicating that the combined treatment effectively alleviated oxidative stress and enhanced the antioxidant defense system of frozen–thawed spermatozoa.

### 3.4. Overview of Metabolomic Profiles

**Figure 1:**
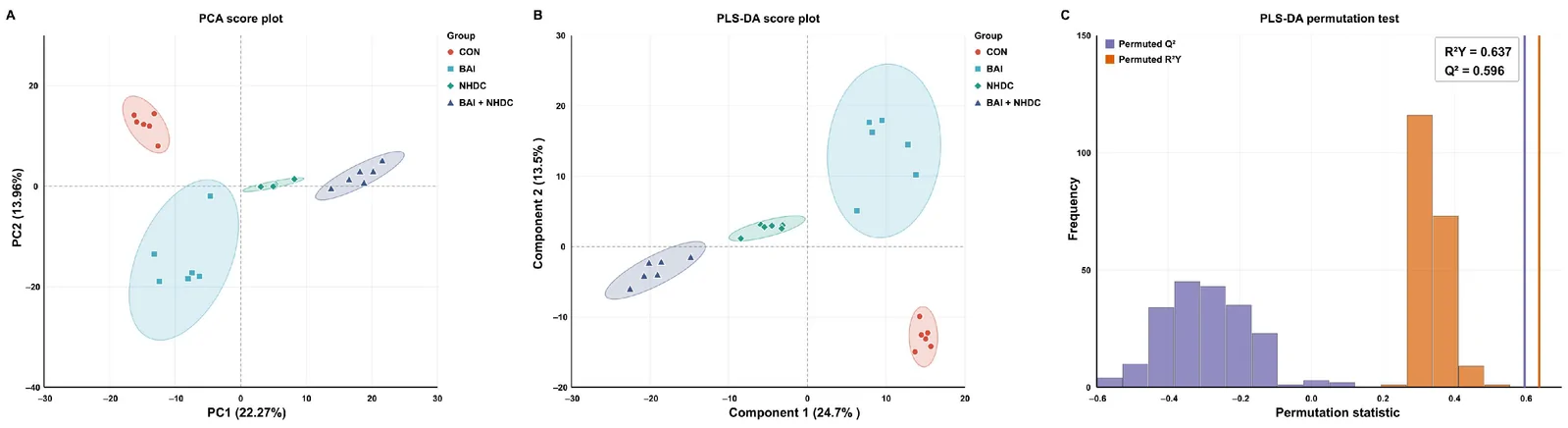
Multivariate analysis of post-thaw semen supernatant metabolomic profiles. (**A**) PCA score plot. (**B**) PLS-DA score plot. (**C**) PLS-DA permutation test.

Metabolomic profiles of post-thaw semen supernatant are shown in Figure 1. PCA indicated separation among the CON, BAI, NHDC and BAI + NHDC groups (PC1 = 22.27%, PC2 = 13.96%; Figure 1A). PLS-DA further showed separation among the four groups (Figure 1B). The model yielded R^2^Y = 0.637 and Q^2^ = 0.596, and permutation testing supported the robustness of the model (Figure 1C).

### 3.5. Differential Metabolites and Enriched Pathways

**Figure 2:**
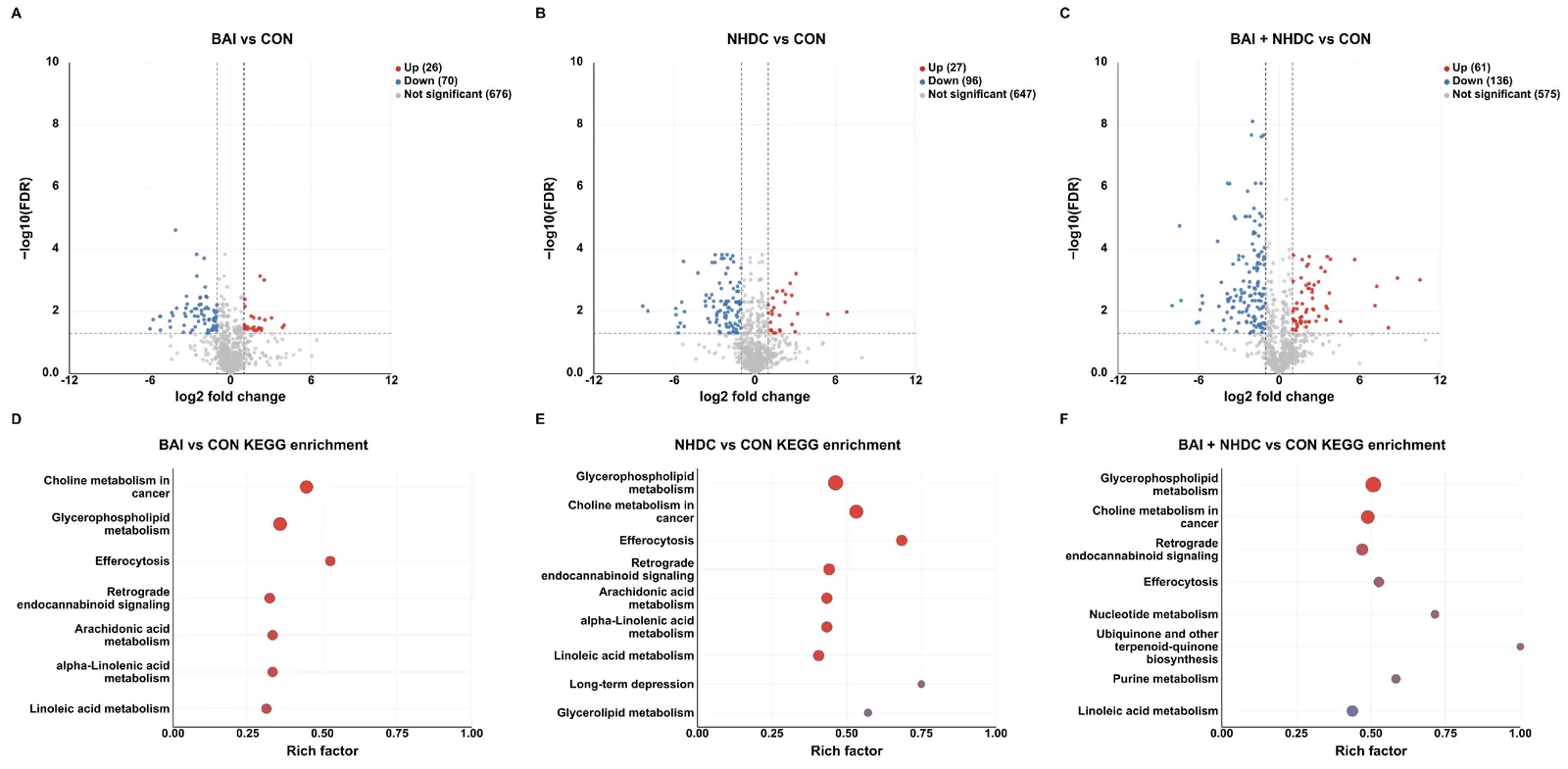
Differential metabolites and KEGG pathway enrichment in the BAI, NHDC and BAI + NHDC groups compared with CON. (**A**–**C**) Volcano plots of BAI vs. CON, NHDC vs. CON and BAI + NHDC vs. CON, respectively. (**D**–**F**) KEGG enrichment bubble plots of the corresponding differential metabolites.

Differential metabolites and enriched pathways are shown in Figure 2. Compared with CON, 96 differential metabolites were identified in the BAI group, including 26 up-regulated and 70 down-regulated metabolites (Figure 2A). In the NHDC group, 123 differential metabolites were identified, including 27 up-regulated and 96 down-regulated metabolites (Figure 2B). In the BAI + NHDC group, 197 differential metabolites were identified, including 61 up-regulated and 136 down-regulated metabolites (Figure 2C). KEGG enrichment analysis identified 7, 9 and 8 enriched pathways in the BAI, NHDC and BAI + NHDC groups, respectively (Figure 2D–F). The top 20 differential metabolites in BAI vs. CON, NHDC vs. CON, and BAI + NHDC vs. CON are summarized in Supplementary Tables S1–S3, respectively.

### 3.6. Shared Differential Metabolites Among the Three Comparisons

**Figure 3:**
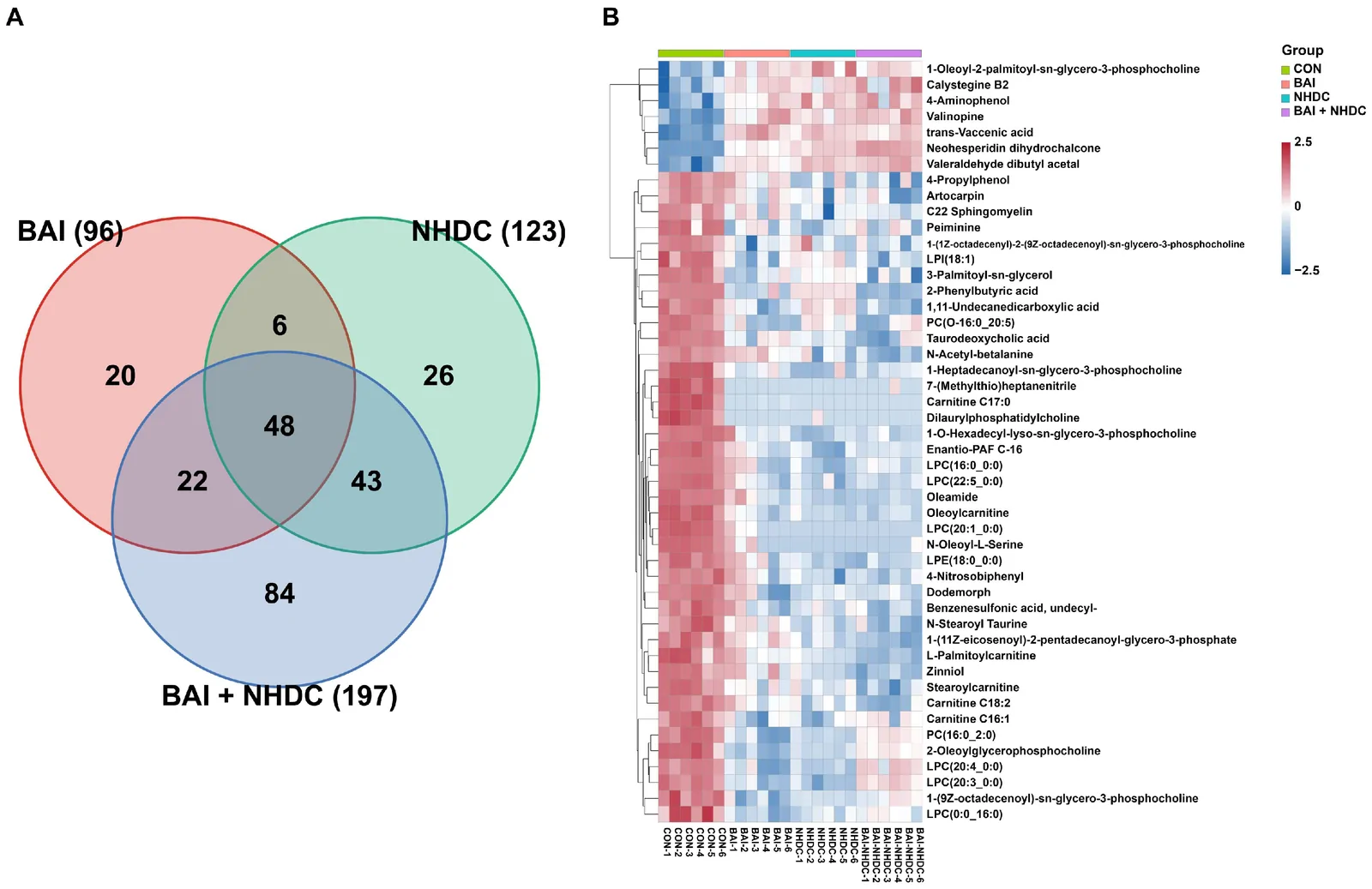
Shared differential metabolites among BAI, NHDC and BAI + NHDC treatments. (**A**) Venn diagram of differential metabolites in BAI vs. CON, NHDC vs. CON and BAI + NHDC vs. CON. (**B**) Heatmap of the 48 shared differential metabolites.

Shared differential metabolites are shown in Figure 3. A total of 48 metabolites were shared among BAI vs. CON, NHDC vs. CON and BAI + NHDC vs. CON (Figure 3A). Most shared metabolites showed the same direction of change across the three treatment groups. These shared metabolites showed distinct abundance patterns across the four groups and included multiple metabolite classes, including organic acids and derivatives, lipids and lipid-like molecules, organic nitrogen compounds and organoheterocyclic compounds (Figure 3B). The 48 shared differential metabolites and their directions of change are listed in Supplementary Table S4.

### 3.7. BAI × NHDC Interaction-Associated Metabolites

**Figure 4:**
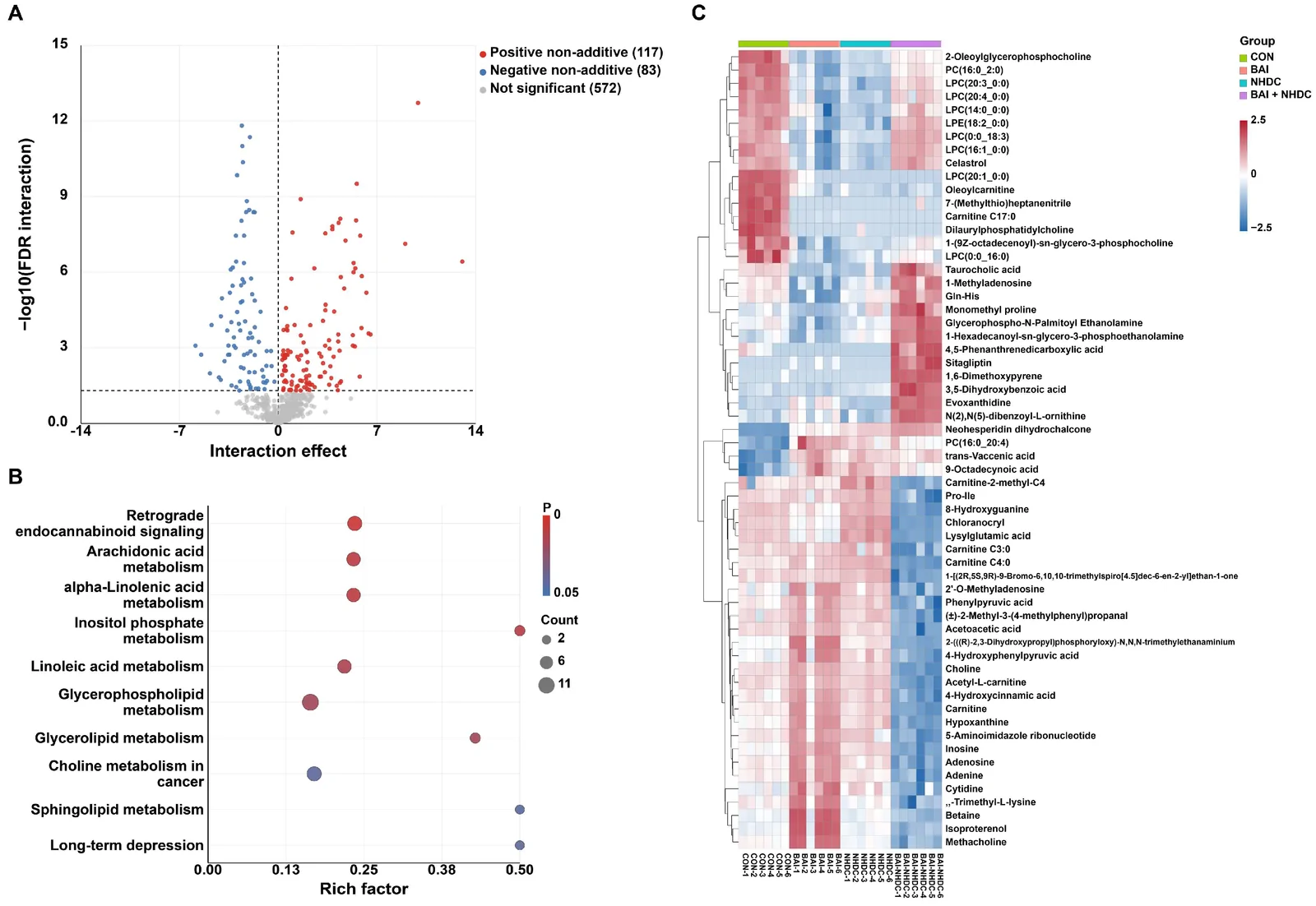
BAI × NHDC interaction-associated metabolites. (**A**) Volcano plot of BAI × NHDC interaction-associated metabolites. (**B**) KEGG enrichment bubble plot of interaction-associated metabolites. (**C**) Heatmap of interaction-associated metabolites.

BAI × NHDC interaction-associated metabolites are shown in Figure 4. A total of 200 metabolites were retained as BAI × NHDC interaction-associated metabolites, including 117 positive non-additive and 83 negative non-additive metabolites (Figure 4A). KEGG enrichment analysis showed that these metabolites were mainly enriched in glycerophospholipid metabolism, linoleic acid metabolism, α-linolenic acid metabolism, arachidonic acid metabolism, retrograde endocannabinoid signaling and inositol phosphate metabolism (Figure 4B). The interaction-associated metabolites showed distinct abundance patterns across the four groups (Figure 4C). The top 20 BAI × NHDC interaction-associated metabolites and their statistical results are presented in Supplementary Table S5.

## 4. Discussion

Semen cryopreservation exposes sperm to multiple stressors, including cold shock, ice crystal formation, osmotic fluctuation, and oxidative stress [3,24,25]. Among these, oxidative stress is an important cause of reduced post-thaw sperm quality [26]. The freeze–thaw process induces ROS accumulation, disrupts redox balance and damages the sperm plasma membrane, acrosome and mitochondria [27]. In the present study, BAI, NHDC and BAI + NHDC improved post-thaw motility, plasma membrane integrity, acrosome integrity, ATP content and MMP in Qinchuan bull sperm. They also reduced ROS and MDA levels and increased T-AOC, SOD, CAT and GSH-Px activities. These results indicate that BAI and NHDC enhanced antioxidant defense in post-thaw sperm and that the combination provided greater antioxidant protection. Control differences among the three independent experiments may reflect batch-to-batch variation in semen quality and cryopreservation response, potentially influenced by seasonal factors [28]. Furthermore, total motility increased more than progressive motility, suggesting a contribution from non-progressive motility.

This protection was associated with reduced oxidative damage. The sperm plasma and acrosomal membranes are rich in polyunsaturated fatty acids and are highly sensitive to oxidative stress [29]. Excess ROS can induce lipid peroxidation and impair membrane integrity and fluidity [30]. Mitochondria are the main sites of ATP synthesis, and mitochondrial damage can further promote ROS generation [31]. BAI, with its vicinal trihydroxyl flavone structure, efficiently scavenges hydroxyl and peroxyl radicals, thereby limiting ROS accumulation and alleviating oxidative damage [32]. NHDC, as a dihydrochalcone compound, also has strong radical-scavenging activity and can improve antioxidant capacity [33,34]. Their antioxidant effects may also involve direct radical scavenging and protection of endogenous antioxidant enzymes from oxidative inactivation. In the present study, BAI and NHDC each exhibited substantial antioxidant capacity, protecting sperm membrane architecture and mitochondrial status through enhanced antioxidant defense and reduced lipid peroxidation, consistent with previous reports [13]. The reduced benefits at higher concentrations may reflect a concentration-dependent balance between antioxidant protection and adverse effects on sperm function. Pro-oxidant activity and mitochondrial disturbances have been reported for several polyphenols in human spermatozoa [35], although these mechanisms were not directly established in the present study. However, direct contributions of BAI and NHDC to the colorimetric assay readouts cannot be excluded in the absence of cell-free controls.

Metabolomic analysis provided further insight into these phenotypic changes. Differential metabolites from the three treatments were consistently enriched in glycerophospholipid and linoleic acid metabolism, while BAI and NHDC also showed enrichment of α-linolenic acid and arachidonic acid metabolism. These pathways involve membrane phospholipid remodeling and unsaturated fatty acid metabolism, processes relevant to sperm membrane stability and lipid peroxidation [36,37]. These metabolic changes were consistent with the higher plasma membrane and acrosome integrity and lower MDA levels observed in the treatment groups. However, KEGG categories such as “Choline metabolism in cancer,” “Efferocytosis,” and “Long-term depression” mainly reflect shared lipid-related metabolite annotations and do not indicate the occurrence of these biological processes in spermatozoa.

The treatment groups also showed distinct metabolic responses. The NHDC group was specifically enriched in glycerolipid metabolism, suggesting alterations in lipid turnover and membrane lipid precursor availability [38]. In contrast, the BAI + NHDC group was specifically enriched in nucleotide metabolism, purine metabolism, and ubiquinone and other terpenoid–quinone biosynthesis. These pathways are closely associated with nucleotide turnover, mitochondrial energy metabolism, and electron transport [39,40], which may be related to the changes in ATP content and mitochondrial function observed after treatment.

The analysis of the 48 shared differential metabolites indicated a common core metabolic response among the three treatment groups. These metabolites were mainly associated with membrane phospholipid turnover and fatty acid-related energy metabolism. Their concordant changes suggest that BAI and NHDC reduced the accumulation of products related to membrane phospholipid degradation, lipid release, or oxidative membrane injury after freezing and thawing [41]. These metabolic alterations were consistent with the higher plasma membrane and acrosome integrity and lower MDA levels observed in the treatment groups.

The BAI × NHDC interaction analysis further demonstrated that the combined treatment induced non-additive metabolic responses. The interaction-associated metabolites were enriched in lipid-related pathways, including glycerophospholipid metabolism and linoleic acid metabolism. At the metabolite level, acylcarnitines and purine-related metabolites, including inosine and hypoxanthine, showed non-additive responses. Acylcarnitines are important intermediates in mitochondrial fatty acid transport and energy metabolism [42]. The altered acylcarnitine profile suggests that the combined treatment may influence mitochondrial substrate utilization after thawing [43]. In addition, changes in purine-related metabolites, including inosine and hypoxanthine, may reflect alterations in nucleotide turnover and energy metabolism [44]. Changes in choline and lysophospholipid-related metabolites indicate that BAI + NHDC may also regulate membrane lipid remodeling processes [41,45].

Overall, the improvements in post-thaw sperm quality following BAI and NHDC supplementation were accompanied by changes in oxidative status, mitochondrial function, and lipid- and energy-related metabolites. The combined treatment was associated with non-additive changes in acylcarnitine-, purine-, and membrane lipid-related metabolites. These findings suggest that BAI and NHDC may improve post-thaw sperm quality through antioxidant effects and changes in lipid- and energy-related metabolism, providing a rationale for further optimization of bull semen freezing extenders. Untargeted metabolomics was exploratory, and the absence of a cell-free extender control prevented separation of extender- and additive-derived contributions from sperm-associated changes. The higher number of differential metabolites in the BAI + NHDC group may partly reflect additive-derived signals and does not, by itself, demonstrate stronger regulation of endogenous sperm metabolism. DNA integrity, capacitation status, fertilization capacity, and fertility were not assessed in this study. Therefore, targeted and functional validation will be carried out in future studies to determine the roles of the key metabolite annotations and their biological relevance.

## 5. Conclusions

In conclusion, BAI, NHDC, and their combination improved the post-thaw quality of Qinchuan bull sperm. The supplemented groups showed higher plasma membrane integrity, acrosome integrity, ATP content, and MMP, together with higher antioxidant enzyme activities and T-AOC. The combined treatment showed higher total motility, plasma membrane integrity, and acrosome integrity than either single treatment. Metabolomic results suggest that these effects were associated with reduced oxidative stress, maintained mitochondrial function, and changes in lipid- and energy-related metabolism. The non-additive metabolic response induced by the combined treatment suggests that BAI + NHDC interacted at the metabolic level during cryopreservation.

## Figures and Tables

**Table 1:** Effects of BAI, NHDC, and BAI + NHDC on Post-Thaw Sperm Motility Parameters.

Group	Concentrations (μg/mL)	Total Motility (%)	Progressive Motility (%)	SQA-Vb-Reported Velocity (μm/s)
BAI	0	42.67 ± 1.59 ^d^	37.21 ± 0.93 ^d^	35.75 ± 2.69 ^c^
	20	56.88 ± 3.08 ^b^	39.30 ± 0.71 ^bc^	38.18 ± 1.39 ^bc^
	40	61.78 ± 1.63 ^a^	41.18 ± 0.49 ^a^	41.74 ± 1.42 ^a^
	60	56.32 ± 2.24 ^b^	40.18 ± 0.49 ^b^	39.87 ± 1.54 ^ab^
	80	51.18 ± 2.05 ^c^	38.62 ± 0.61 ^c^	38.53 ± 1.35 ^ab^
NHDC	0	43.12 ± 1.23 ^d^	38.39 ± 0.58 ^b^	31.80 ± 1.17 ^c^
	10	52.42 ± 1.59 ^c^	38.75 ± 0.60 ^b^	34.44 ± 1.12 ^b^
	20	57.59 ± 1.66 ^b^	39.42 ± 0.51 ^b^	34.46 ± 1.09 ^b^
	30	62.69 ± 1.34 ^a^	41.68 ± 0.70 ^a^	37.42 ± 1.61 ^a^
	40	59.24 ± 0.96 ^b^	39.49 ± 0.91 ^b^	34.83 ± 0.83 ^b^
BAI + NHDC	CON	55.80 ± 1.20 ^c^	38.67 ± 0.74 ^b^	33.03 ± 0.59 ^c^
	BAI (40 μg/mL)	61.20 ± 0.96 ^b^	40.96 ± 0.74 ^a^	39.86 ± 1.01 ^b^
	NHDC (30 μg/mL)	62.08 ± 0.70 ^b^	41.35 ± 0.70 ^a^	40.35 ± 0.68 ^ab^
	BAI + NHDC	64.93 ± 1.31 ^a^	41.98 ± 0.79 ^a^	41.49 ± 0.70 ^a^

Values are mean ± SD (n = 6). Different superscript letters within the same experimental block and column indicate significant differences (*p* < 0.05). Data from the BAI screening, NHDC screening, and four-group comparison were analyzed separately. BAI + NHDC contained 40 μg/mL BAI and 30 μg/mL NHDC.

**Table 2:** Effects of BAI, NHDC and BAI + NHDC on post-thaw structural integrity and mitochondrial function.

Group	Concentrations (μg/mL)	Membrane Integrity (%)	Acrosome Integrity (%)	ATP (μmol/mL)	MMP (Red/Green Fluorescence Ratio)
BAI	0	41.17 ± 2.61 ^c^	57.60 ± 1.96 ^c^	0.49 ± 0.07 ^b^	0.81 ± 0.01 ^c^
	20	45.23 ± 2.94 ^b^	61.88 ± 2.08 ^b^	0.60 ± 0.05 ^a^	0.83 ± 0.01 ^b^
	40	52.49 ± 1.19 ^a^	66.99 ± 1.29 ^a^	0.61 ± 0.07 ^a^	0.85 ± 0.01 ^a^
	60	49.14 ± 2.47 ^ab^	60.57 ± 1.15 ^b^	0.57 ± 0.07 ^ab^	0.83 ± 0.01 ^b^
	80	47.32 ± 2.90 ^b^	57.30 ± 3.78 ^c^	0.55 ± 0.05 ^ab^	0.83 ± 0.01 ^b^
NHDC	0	41.66 ± 2.71 ^d^	53.42 ± 1.58 ^c^	0.44 ± 0.04 ^b^	0.81 ± 0.01 ^c^
	10	46.39 ± 1.33 ^c^	55.77 ± 1.67 ^c^	0.63 ± 0.05 ^a^	0.82 ± 0.01 ^bc^
	20	49.89 ± 1.00 ^b^	59.06 ± 1.13 ^b^	0.63 ± 0.06 ^a^	0.84 ± 0.01 ^b^
	30	52.61 ± 1.17 ^a^	63.41 ± 1.86 ^a^	0.60 ± 0.06 ^a^	0.87 ± 0.02 ^a^
	40	49.30 ± 1.47 ^b^	61.06 ± 1.13 ^ab^	0.54 ± 0.04 ^a^	0.83 ± 0.01 ^b^
BAI + NHDC	CON	44.37 ± 1.07 ^c^	56.03 ± 1.26 ^d^	0.43 ± 0.06 ^c^	0.72 ± 0.01 ^c^
	BAI (40 μg/mL)	49.67 ± 1.84 ^b^	62.99 ± 1.80 ^c^	0.60 ± 0.01 ^b^	0.84 ± 0.02 ^b^
	NHDC (30 μg/mL)	50.83 ± 0.59 ^b^	66.51 ± 2.18 ^b^	0.65 ± 0.03 ^ab^	0.88 ± 0.02 ^a^
	BAI + NHDC	53.74 ± 1.42 ^a^	70.37 ± 1.65 ^a^	0.68 ± 0.02 ^a^	0.92 ± 0.02 ^a^

Values are mean ± SD (n = 6). Different superscript letters within the same experimental block and column indicate significant differences (*p* < 0.05). Data from the BAI screening, NHDC screening, and four-group comparison were analyzed separately. BAI + NHDC contained 40 μg/mL BAI and 30 μg/mL NHDC.

**Table 3:** Effects of BAI, NHDC and BAI + NHDC on post-thaw oxidative status and antioxidant capacity.

Group	Concentrations (μg/mL)	Relative ROS Level	SOD (U/mL)	CAT (U/mL)	GSH-Px (U/mL)	T-AOC (μmol/mL)	MDA (nmol/mL)
BAI	0	0.92 ± 0.03 ^a^	55.25 ± 2.59 ^d^	3.01 ± 0.54 ^c^	158.87 ± 10.55 ^c^	2.87 ± 0.07 ^e^	6.15 ± 0.54 ^a^
	20	0.85 ± 0.04 ^b^	92.56 ± 3.21 ^b^	5.52 ± 1.16 ^b^	230.24 ± 46.11 ^c^	3.63 ± 0.06 ^b^	5.34 ± 0.15 ^b^
	40	0.76 ± 0.02 ^c^	102.99 ± 2.69 ^a^	7.39 ± 1.34 ^a^	378.80 ± 63.54 ^a^	3.78 ± 0.07 ^a^	4.16 ± 0.31 ^c^
	60	0.87 ± 0.03 ^ab^	98.01 ± 2.58 ^a^	4.50 ± 0.40 ^bc^	337.90 ± 71.84 ^ab^	3.49 ± 0.06 ^c^	4.83 ± 0.31 ^b^
	80	0.84 ± 0.02 ^b^	83.01 ± 2.62 ^c^	3.61 ± 0.63 ^c^	249.32 ± 54.70 ^bc^	3.26 ± 0.05 ^d^	5.33 ± 0.26 ^b^
NHDC	0	0.97 ± 0.03 ^a^	70.08 ± 1.83 ^d^	2.88 ± 0.32 ^d^	119.84 ± 10.08 ^d^	2.82 ± 0.05 ^d^	6.02 ± 0.58 ^a^
	10	0.92 ± 0.03 ^b^	83.38 ± 5.76 ^c^	3.65 ± 0.16 ^d^	216.94 ± 28.62 ^c^	2.97 ± 0.08 ^c^	5.13 ± 0.16 ^b^
	20	0.86 ± 0.03 ^c^	91.71 ± 1.75 ^b^	6.41 ± 0.76 ^b^	319.67 ± 25.02 ^ab^	3.15 ± 0.03 ^b^	4.77 ± 0.18 ^bc^
	30	0.80 ± 0.02 ^d^	102.33 ± 2.78 ^a^	7.88 ± 0.88 ^a^	346.89 ± 39.21 ^a^	3.45 ± 0.07 ^a^	4.24 ± 0.16 ^c^
	40	0.88 ± 0.02 ^bc^	77.96 ± 2.77 ^c^	5.92 ± 0.37 ^c^	259.15 ± 47.85 ^bc^	3.08 ± 0.04 ^b^	5.46 ± 0.35 ^ab^
BAI + NHDC	CON	0.91 ± 0.02 ^a^	59.21 ± 1.86 ^d^	2.71 ± 0.50 ^d^	178.84 ± 9.27 ^d^	2.93 ± 0.14 ^d^	6.14 ± 0.37 ^a^
	BAI (40 μg/mL)	0.81 ± 0.07 ^b^	83.69 ± 4.42 ^c^	7.21 ± 0.65 ^c^	249.28 ± 5.94 ^c^	3.39 ± 0.04 ^c^	4.99 ± 0.28 ^b^
	NHDC (30 μg/mL)	0.77 ± 0.11 ^b^	93.52 ± 2.72 ^b^	8.85 ± 0.31 ^b^	284.12 ± 23.39 ^b^	3.69 ± 0.19 ^b^	4.52 ± 0.13 ^c^
	BAI + NHDC	0.68 ± 0.05 ^c^	101.57 ± 1.47 ^a^	10.64 ± 1.56 ^a^	332.66 ± 19.10 ^a^	3.96 ± 0.18 ^a^	4.04 ± 0.15 ^d^

Values are mean ± SD (n = 6). Different superscript letters within the same experimental block and column indicate significant differences (*p* < 0.05). Data from the BAI screening, NHDC screening, and four-group comparison were analyzed separately. BAI + NHDC contained 40 μg/mL BAI and 30 μg/mL NHDC.

## Data Availability

The raw metabolomics data generated in this study have been deposited in the OMIX repository at the National Genomics Data Center (NGDC) under accession number OMIX021547. Other data supporting the conclusions of this study are available from the authors upon request.

## Supplementary Materials

The following supporting information can be downloaded at: https://www.mdpi.com/article/10.3390/ani16203210/s1, Table S1: Top 20 differential metabolites in BAI vs. CON; Table S2: Top 20 differential metabolites in NHDC vs. CON; Table S3: Top 20 differential metabolites in BAI + NHDC vs. CON; Table S4: The 48 shared differential metabolites; Table S5: Top 20 BAI × NHDC interaction-associated metabolites; Figure S1. Diagram of acrosome integrity and plasma membrane integrity. A. Illustrative images of acrosome integrity assessment using FITC-PNA staining. S1 indicates sperm with intact acrosomes, and S2 indicates sperm with damaged or incomplete acrosomes. B. Illustrative image of plasma membrane integrity assessment using the hypo-osmotic swelling test (HOST). Horizontal and vertical arrows indicate sperm classified as having intact and damaged plasma membranes, respectively.

## Abbreviations

The following abbreviations are used in this manuscript:

BAI	baicalein
NHDC	neohesperidin dihydrochalcone
ROS	reactive oxygen species
MDA	malondialdehyde
SOD	superoxide dismutase
CAT	catalase
GSH-Px	glutathione peroxidase
T-AOC	total antioxidant capacity
ATP	adenosine triphosphate
MMP	mitochondrial membrane potential
KEGG	Kyoto Encyclopedia of Genes and Genomes
