# Supplementary material for: Baicalein and Neohesperidin Dihydrochalcone Improve Post-Thaw Sperm Quality and Modulate the Semen Supernatant Metabolome in Qinchuan Bulls
Source: Animals (Basel). 2026 Oct 12;16(20):3210. doi: 10.3390/ani16203210 (PMC13700661; doi:10.3390/ani16203210)
Supplement: Supplementary file 1 [file animals-16-03210-s001.zip › animals-4570004-supplementary.pdf]

Supplementary Table S1. Top 20 differential metabolites in BAI vs CON.

Metabolite	Direction	log2FC	FDR	VIP
2-Phenylbutyric acid	Down	-4.101	2.40E−05	1.650
Taurodeoxycholic acid	Down	-2.526	1.44E−04	1.604
Celastrol	Down	-1.969	1.94E−04	1.663
Valeraldehyde dibutyl acetal	Up	2.197	7.17E−04	1.570
LPC (16:0_0:0)	Down	-2.510	7.17E−04	1.506
Glucosamine	Up	2.503	9.69E−04	1.795
LPC (14:0_0:0)	Down	-1.884	1.62E−03	1.439
LPC (20:4_0:0)	Down	-3.283	3.23E−03	1.623
1-Methyladenosine	Down	-1.832	3.23E−03	1.519
LPC (20:3_0:0)	Down	-2.246	3.46E−03	1.711
Oleamide	Down	-1.759	3.46E−03	1.552
Carnitine C18:2	Down	-2.280	3.65E−03	1.605
4-Aminophenol	Up	1.074	3.97E−03	1.544
PC (16:0_2:0)	Down	-3.225	5.76E−03	1.687
1,11-Undecanedicarboxylic acid	Down	-2.201	5.88E−03	1.602
LPC (16:1_0:0)	Down	-2.332	6.07E−03	1.664
N-Acetyl-betalanine	Down	-1.030	6.14E−03	1.521
Calystegine B2	Up	1.082	6.84E−03	1.450
LPE (18:0_0:0)	Down	-1.784	7.26E−03	1.445
Oleoylcarnitine	Down	-3.325	7.72E−03	1.713

Supplementary Table S2. Top 20 differential metabolites in NHDC vs CON.

Metabolite	Direction	log2FC	FDR	VIP
Dodemorph	Down	-2.077	1.47E−04	1.659
N-(1-Methyl-5-indolyl)-N'-(3-pyridyl) urea	Down	-2.447	1.47E−04	1.537
2-Phenylbutyric acid	Down	-2.953	1.48E−04	1.689
N-Acetylserotonin	Down	-1.646	1.61E−04	1.579
LPC (14:0_0:0)	Down	-2.485	1.95E−04	1.674
Acetaminophen	Down	-2.319	1.95E−04	1.473
Celastrol	Down	-1.800	2.02E−04	1.640
1-O-Hexadecyl-lyso-sn-glycero-3-phosphocholine	Down	-5.312	2.43E−04	1.661
10-hydroxystearic acid	Down	-1.582	2.51E−04	1.546
Oleamide	Down	-2.967	2.61E−04	1.665
Taurodeoxycholic acid	Down	-3.189	2.61E−04	1.634
Myristic acid	Down	-1.020	3.95E−04	1.657
N-Acetyl-betalanine	Down	-2.019	3.95E−04	1.415
LPC (16:0_0:0)	Down	-4.245	5.70E−04	1.639
Neohesperidin dihydrochalcone	Up	3.073	6.09E−04	1.685
1-Stearoyl-2-myristoyl-sn-glycero-3-phosphocholine	Down	-2.171	6.09E−04	1.546
Dodecanal dimethyl acetal	Down	-1.143	1.15E−03	1.664
6-Dimethylaminopurine	Down	-1.824	1.15E−03	1.506
3-Methoxy-L-tyrosine	Down	-2.631	1.22E−03	1.521
Valeraldehyde dibutyl acetal	Up	2.639	1.23E−03	1.519

Supplementary Table S3. Top 20 differential metabolites in BAI + NHDC vs CON.

Metabolite	Direction	log2FC	FDR	VIP
Choline	Down	-1.982	7.70E−09	1.527
Chloranocryl	Down	-2.063	2.12E−08	1.537
2-(((R)-2,3-Dihydroxypropyl) phosphoryloxy)-N, N, N-trimethylethanaminium	Down	-1.153	2.12E−08	1.533
Carnitine	Down	-1.320	2.40E−08	1.526
Carnitine C4:0	Down	-1.761	7.51E−07	1.537
Acetyl-L-carnitine	Down	-1.346	7.51E−07	1.508
8-Hydroxyguanosine	Down	-3.832	7.51E−07	1.470
3,4-Bis[(3-hydroxyphenyl) methyl] oxolan-2-one	Down	-3.685	7.64E−07	1.429
5-Aminoimidazole ribonucleotide	Down	-2.350	1.35E−06	1.486
Adenosine	Down	-1.886	4.74E−06	1.478
Hypoxanthine	Down	-1.438	7.05E−06	1.508
Lysylglutamic acid	Down	-2.498	8.76E−06	1.540
8-Hydroxyguanine	Down	-3.349	8.76E−06	1.533
Dodemorph	Down	-2.178	8.76E−06	1.512
Inosine	Down	-1.291	8.76E−06	1.493
Carnitine C3:0	Down	-2.470	8.76E−06	1.438
Taurodeoxycholic acid	Down	-3.260	1.03E−05	1.357
Adenine	Down	-1.809	1.23E−05	1.423
Methacholine	Down	-1.437	1.64E−05	1.444
2-Phenylbutyric acid	Down	-7.421	1.78E−05	1.537

Supplementary Table S4. The 48 shared differential metabolites.

Metabolite	Direction pattern
LPE (18:0_0:0)	down/down/down
1-(9Z-octadecenoyl)-sn-glycero-3-phosphocholine	down/down/down
1-Heptadecanoyl-sn-glycero-3-phosphocholine	down/down/down
LPC (20:4_0:0)	down/down/down
LPC (20:3_0:0)	down/down/down
PC(O-16:0_20:5)	down/down/down
1-O-Hexadecyl-lyso-sn-glycero-3-phosphocholine	down/down/down
C22 Sphingomyelin	down/down/down
1-(1Z-octadecenyl)-2-(9Z-octadecenoyl)-sn-glycero-3-phosphocholine	down/down/down
LPC (22:5_0:0)	down/down/down
Dilaurylphosphatidylcholine	down/down/down
LPC (20:1_0:0)	down/down/down
Enantio-PAF C-16	down/down/down
PC (16:0_2:0)	down/down/down
LPC (16:0_0:0)	down/down/down
2-Oleoylglycerophosphocholine	down/down/down
LPC (0:0_16:0)	down/down/down
1-Oleoyl-2-palmitoyl-sn-glycero-3-phosphocholine	up/up/up
1-(11Z-eicosenoyl)-2-pentadecanoyl-glycero-3-phosphate	down/down/down
LPI (18:1)	down/down/down
Oleoylcarnitine	down/down/down
Stearoylcarnitine	down/down/down
Carnitine C18:2	down/down/down
Carnitine C17:0	down/down/down
Carnitine C16:1	down/down/down
L-Palmitoylcarnitine	down/down/down
3-Palmitoyl-sn-glycerol	down/down/down
Oleamide	down/down/down

1,11-Undecanedicarboxylic acid	down/down/down
trans-Vaccenic acid	up/up/up
N-Stearoyl Taurine	down/down/down
Taurodeoxycholic acid	down/down/down
N-Oleoyl-L-Serine	down/down/down
Valinopine	up/up/up
4-Aminophenol	up/up/up
4-Propylphenol	down/down/down
Neohesperidin dihydrochalcone	up/up/up
7-(Methylthio)heptanenitrile	down/down/down
Dodemorph	down/down/down
4-Nitrosobiphenyl	down/down/down
Benzenesulfonic acid, undecyl-	down/down/down
Valeraldehyde dibutyl acetal	up/up/up
Peiminine	down/down/down
Artocarpin	down/down/down
Calystegine B2	up/up/up
2-Phenylbutyric acid	down/down/down
N-Acetyl-betalanine	down/down/down
Zinniol	down/down/down

Interaction effects were calculated using $\log_2$-transformed metabolite intensities as mean (BAI + NHDC) − mean (BAI) − mean (NHDC) + mean (CON). Positive and negative values indicate deviations above and below the additive expectation on this scale, respectively.

Supplementary Table S5. Top 20 BAI × NHDC interaction-associated metabolites.

Metabolite	Interaction type	Interaction effect	*P* interaction	FDR interaction
1,6-Dimethoxypyrene	Positive non-additive	9.914	2.47E-16	1.91E-13
Carnitine C4:0	Negative non-additive	-2.586	3.96E-15	1.53E-12
Chloranocryl	Negative non-additive	-2.013	1.69E-14	4.35E-12
Inosine	Negative non-additive	-2.548	5.11E-14	9.86E-12
Choline	Negative non-additive	-2.509	2.81E-13	4.34E-11
8-Hydroxyguanine	Negative non-additive	-2.921	1.10E-12	1.42E-10
Carnitine C17:0	Positive non-additive	5.567	2.80E-12	3.09E-10
3,5-Dihydroxybenzoic acid	Positive non-additive	1.586	1.30E-11	1.26E-09
Lysylglutamic acid	Negative non-additive	-2.232	1.76E-11	1.51E-09
Carnitine	Negative non-additive	-2.062	4.36E-11	3.36E-09
Neohesperidin dihydrochalcone	Negative non-additive	-1.756	6.01E-11	4.15E-09
1-[(2R,5S,9R)-9-Bromo-6,10,10-trimethylspiro [4.5] dec-6-en-2-yl] ethan-1-one	Negative non-additive	-2.279	6.44E-11	4.15E-09
Acetyl-L-carnitine	Negative non-additive	-1.679	7.07E-11	4.20E-09
LPC (16:1_0:0)	Positive non-additive	4.398	1.37E-10	7.56E-09
7-(Methylthio)heptanenitrile	Positive non-additive	5.517	1.72E-10	8.83E-09
Hypoxanthine	Negative non-additive	-2.607	1.89E-10	9.13E-09
2-Oleoylglycerophosphocholine	Positive non-additive	4.276	2.43E-10	1.10E-08
LPC (20:3_0:0)	Positive non-additive	3.842	3.52E-10	1.51E-08
Celastrol	Positive non-additive	3.840	4.87E-10	1.98E-08
Taurocholic acid	Positive non-additive	1.007	6.88E-10	2.65E-08

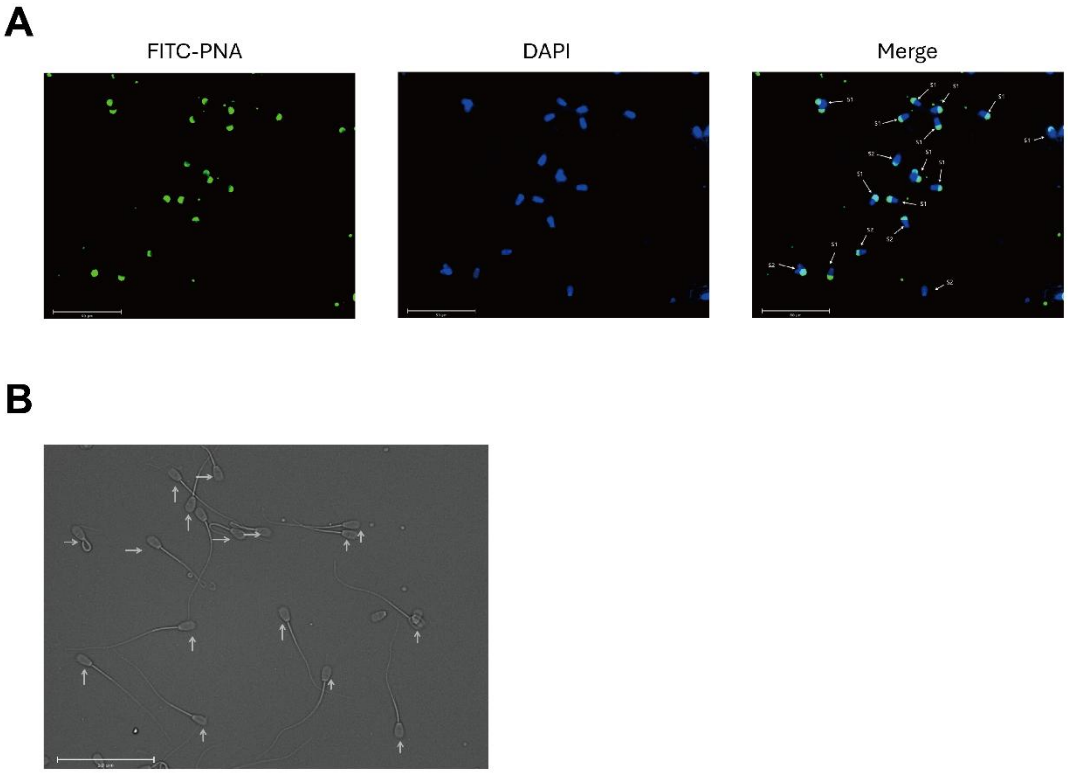

Figure S1. Diagram of acrosome integrity and plasma membrane integrity**. A.** Illustrative images of acrosome integrity assessment using FITC-PNA staining. S1 indicates sperm with intact acrosomes, and S2 indicates sperm with damaged or incomplete acrosomes.**B.** Illustrative image of plasma membrane integrity assessment using the hypo-osmotic swelling test (HOST). Horizontal and vertical arrows indicate sperm classified as having intact and damaged plasma membranes, respectively.
